# Improving neurophysiological biomarkers for functional myoclonic movements

**DOI:** 10.1016/j.parkreldis.2018.03.029

**Published:** 2018-06

**Authors:** M. Beudel, R. Zutt, A.M. Meppelink, S. Little, J.W. Elting, B.M.L. Stelten, M. Edwards, M.A.J. Tijssen

**Affiliations:** a University Groningen, University Medical Center Groningen, Department of Neurology, NL-9700 RB, Groningen, The Netherlands; b Department of Neurology, Haga Teaching Hospital, The Hague, The Netherlands; c Sobell Department of Motor Neuroscience and Movement Disorders, Institute of Neurology, Queen Square, London, UK; d Canisius-Wilhelmina Hospital, Department of Neurology, Nijmegen, The Netherlands; e Institute of Molecular and Clinical Sciences, St George's University of London, London, UK

**Keywords:** Neurophysiological biomarkers, Bereitschaftspotential, Event related desynchronisation, Functional myoclonic jerks, BP, Bereitschaftspotential, CM, cortical myoclonus, ERD, Event Related Desynchronisation, FJ, functional jerks

## Abstract

**Introduction:**

Differentiating between functional jerks (FJ) and organic myoclonus can be challenging. At present, the only advanced diagnostic biomarker to support FJ is the Bereitschaftspotential (BP). However, its sensitivity is limited and its evaluation subjective. Recently, event related desynchronisation in the broad beta range (13–45 Hz) prior to functional generalised axial (propriospinal) myoclonus was reported as a possible complementary diagnostic marker for FJ. Here we study the value of ERD together with a quantified BP in clinical practice.

**Methods:**

Twenty-nine patients with FJ and 16 patients with cortical myoclonus (CM) were included. Jerk-locked back-averaging for determination of the ‘classical’ and quantified BP, and time-frequency decomposition for the event related desynchronisation (ERD) were performed. Diagnostic gain, sensitivity and specificity were obtained for individual and combined techniques.

**Results:**

We detected a classical BP in 14/29, a quantitative BP in 15/29 and an ERD in 18/29 patients. At group level we demonstrate that ERD in the broad beta band preceding a jerk has significantly higher amplitude in FJ compared to CM (respectively −0.14 ± 0.13 and +0.04 ± 0.09 (*p* < 0.001)). Adding ERD to the classical BP achieved an additional diagnostic gain of 53%. Furthermore, when combining ERD with quantified and classical BP, an additional diagnostic gain of 71% was achieved without loss of specificity.

**Conclusion:**

Based on the current findings we propose to the use of combined beta ERD assessment and quantitative BP analyses in patients with a clinical suspicion for all types of FJ with a negative classical BP.

## Highlights

• Differentiating between functional jerks and organic myoclonus is often difficult.
• The sensitivity of a positive bereitschaftspotential for diagnosing FJ is low.
• FJ are preceded by event related desynchronisation (ERD) in the beta band.
• Combining beta ERD and BP improves diagnosing FJ.
• The ERD method is of special relevance in possible FJ patients with a negative BP.

## 1 Introduction

Myoclonus is a common hyperkinetic movement disorder characterized by sudden involuntary muscle contractions (positive myoclonus) [10] or interruption of muscle activity (negative myoclonus) [17]. Approximately 30–50% of patients presenting with myoclonus are diagnosed with functional (psychogenic) myoclonic jerks (FJ) [26,27]. FJ are clinically characterized by an acute onset and occurrence in rest in particular in a supine position. FJ can be difficult to distinguish from organic myoclonus in clinical practice [24]. This is of crucial importance given different aetiologies, treatment and prognosis [8]. Ideally, the diagnosis of FJ would be supported by sensitive *and* specific diagnostic tests, enabling a “laboratory supported” level of diagnostic certainty [11]. At present, the electrophysiological diagnosis of FJ is based on polymyographic findings (e.g. variable muscle recruitment, variable burst duration of >100 ms, and distractibility/entrainment) and the presence of a Bereitschaftspotential (BP) in the EEG prior to a jerky movement with the advanced technique of Back-averaging. However, the reported sensitivity of a positive BP in FJ is heterogeneous ranging from 25% [18] to more than 80% in selected cohorts [9,23]. This emphasizes the importance to improve the electrophysiological biomarkers in FJ.

In clinical practice there are no standardised criteria that define the presence of a BP, although some have been proposed in the research setting [23]. Currently, the definition of a BP is “*clear and slow negative electrical shift*” over the central cortical areas, that increases over time 1–2 s before movement onset [26]. However, a quantitative method would seem to be highly desirable to standardize laboratory supported diagnosis of FJ.

Recently, a new EEG marker of functional axial jerks has been proposed: event related desynchronisation (ERD) in the broad beta band [18]. Reductions of beta and low gamma oscillations occur prior to cued and self-paced movement [20] and may reflect changes in self-directed attention, as recently highlighted in a new explanatory model for functional neurological symptoms [7]. A recent study also showed ERD in the beta range prior to (psychogenic) non-epileptic seizures, suggesting applicability to functional neurological symptoms more widely and supporting a unifying pathophysiological model [19].

In the present study we aimed to (1) replicate the findings of the first study on ERD in FJ in a cohort with different FJ phenotypes beyond generalised axial (propriospinal) myoclonus, (2) determine the diagnostic gain, specificity and sensitivity of ERD with both classical (subjective) and objective evaluation of the BP (3) develop a new diagnostic approach by combining the results of ERD and BP.

## 2 Methods

### 2.1 Patients

Participants with a diagnosis of FJ who underwent a combined video-polymyography and EMG-EEG back-averaging as part of their diagnostic work-up between 2006 and 2016, were identified from the database of the neurology department of the University Medical Center in Groningen. Electrophysiological testing included a minimum recording time length of 30 min with the aim to register at least 40 myoclonic jerks. Patients with both a clinical *and* an electrophysiological diagnosis of FJ [13] and CM were included in the study [[25], [26], [27]]. All clinical diagnoses were made by a movement disorder specialist (MT) based on a personal clinical assessment of the patient or the review of the clinical details and videotaped clinical examination. The local ethical committee of the University Medical Center in Groningen confirmed that the study could proceed without formal consent in light of the retrospective and anonymised nature of the data (M14.157933).

The clinical diagnosis of FJ was based on positive criteria including an acute onset, inconsistent distribution (proximal > distal), and reduction with distraction [16]. Electrophysiological criteria for FJ included a long and/or variable burst duration, variable muscle recruitment, distractibility, and the presence of a ‘classical’ BP on back-averaging [3,26]. In this cohort, the classical BP was only present in 14/29 (47%) of the FJ cases. The presence of a BP was not crucial to diagnose these patients with FJ, but ensured a 100% certainty in the FJ diagnosis. The objective BP and ERD analyses were not used for the sub-classification of FJ.

Patients with the clinical and electrophysiological diagnosis of CM were included as a control group. All CM subjects previously participated in a study evaluating the value of electrophysiological testing in determination of the myoclonus subtype (2014–2016) [27]. The diagnosis of CM was based on clinical and electrophysiological features. Clinically, patients suffered from myoclonus with a facial and distal (multi-) focal distribution [16]. Electrophysiological criteria for CM included burst duration of less than 100 ms, presence of negative myoclonus, and a positive pre-myoclonic cortical spike on back-averaging (7/16 (44%)) [26]. With the presence of a cortical spike, the certainty of the CM diagnosis increased to 100% but also without positive back-averaging these cases would have been classified with CM.

### 2.2 BP analysis

In order to compare different methods for estimating BP, the BP was determined using two different approaches. For both approaches the onsets of jerks were obtained using an automated ‘level trigger’ and visually inspected for artefacts plus subsequent rejection if necessary. The first approach was the classical visual inspection approach (‘classical’ BP) and was performed using EEG jerk-locked back-averages that were calculated across events (Brain Vision Analyzer 2.1, Brain Products GmbH, München, Germany). This approach was performed prior to the present study as ‘care as usual’ by treating physicians and used in the sub-classification of FJ patients.

### 2.3 Objective BP analysis

Beyond ‘care as usual’, an objective approach (objective BP), obtaining the amplitude of the deflection prior to the myoclonic jerk, was performed. In line with the literature on the time-course of the BP EEG data was epoched from −1500ms relative to movement onset [4]. All quantitative and statistical analyses were performed with custom written scripts using Matlab R2015a (The Mathworks, Natick, MA, USA). With a view to clinical applicability, the approach was kept as simple as possible and overlapping epochs (i.e. jerks with less than 1500 ms duration in between jerks) were not rejected. However, to minimise this effect, the amplitude of the BP was obtained from the last, and steepest, phase of the BP, called the negativity slope which ranges from −500ms to movement onset [12]. So by not including the slowly rising negativity between −1500ms and −500ms before FJ, the risk of overlapping intervals was reduced. Given the heterogeneous localization of the myoclonic jerks (unilateral, axial, and/or bilateral) within and between patients with FJ, the central (Cz) electrode with T5 and T6 as reference were used for obtaining the objective BP. In healthy volunteers, the amplitude of the BP is largest at this electrode, which roughly detects neural activity from the supplementary motor area [5].

### 2.4 ERD analysis

For the analyses of the ERD, the same time-courses as for the objective BP were used. Power spectral density (PSD) was obtained using a fast Fourier transform using a 200 ms spectrogram with a 100 ms sliding window. For the ERD analyses the interval −1500ms prior to jerk onset was used which covers the timing of the main deflection in the previous report on ERD [18].

Since this ERD occurs earlier than the negativity slope in the BP (−500 ms), the whole interval of −1500ms to jerk onset was used for further analyses. For the quantification in the ‘broad beta band’ a range from 13 to 45 Hz consisting of the beta band (13–30 Hz) and the low gamma (30–45 Hz) was used in line with the literature on ERD in the beta range prior to voluntary movements and the findings of the previous report on ERD [18]. Baseline normalisation was performed to the value of the 200 ms window −1500ms prior to jerk onset. ERD was expressed as a fraction of the 200 ms window around - 1500 ms and therefore the ERD represents the power in the window of analysis divided by the baseline power.

### 2.5 Statistical analyses

Descriptive statistics of the patient characteristics are reported using medians and (interquartile) ranges. For the neurophysiological data, data were checked for normality using Koglomorov-Smirnov tests and expressed in means and standard deviations. For the comparison of the objective BP and ERD between patients with CM and FJ with or with without a subjectively defined BP, two-sample *t-*tests were used. Multiple comparisons were corrected by applying the false discovery rate [2]. The correlation between the objective BP and ERD was performed using Pearson's correlation coefficient. Receiver operating characteristics (ROC) were expressed as area under the curve and mutually compared [14]. To combine the objective BP and ERD in the ROC, a rank between 1 and 45 was assigned to every patient for both the objective BP and the ERD. For each subject, the two ranks were added and divided by two. This resulted in an average rank on the combined diagnostic tests.

Finally, the three different approaches, classical (subjective) and quantitative (objective) BP and ERD, plus their combination were compared. This was done by statistically comparing the sensitivities of the different approaches and their combinations at a specificity level of 100%. When one method was superior to another the difference in sensitivity was expressed in a percentage and named ‘diagnostic gain’. Cutoff values for BP and ERD were obtained from the maximum values seen in the CM group. Different approaches, or their combinations were mutually compared using the Wilcoxon rank-sum test.

## 3 Results

### 3.1 Patients

Forty-seven patients with either FJ or CM were identified, of which two were excluded due to the co-existence of both cortical and subcortical myoclonus subtypes. Forty-five patients were included in the study; 29 patients with FJ (48% female, median age at examination 51 years) and 16 with CM (56% female, median age at examination 28 years). The median number of jerks available for back-averaging was 47 (IQR; 36) in the FJ group and 106 (IQR; 323) in the CM group. The clinical and electrophysiological features of both groups are shown in Table 1.

**Table 1:** Clinical and electrophysiological characteristics of the CM and FJ subgroups. Statistically significant differences using Mann-Whitney *U* test (P < 0.05) are highlighted in bold.

Clinical characteristics		CM (n = 16)	FJ (n = 29)
Gender	male/female	7/9	15/14
Age at examination, median (range)		**28 (6–73)**	**51 (15–77)**
Age at onset of myoclonus median (range)		**22 (4–73)**	**43 (13–75)**
Onset	acute/subacute	5/0	8/11
	gradually	11	5
	missing	0	5
Preceding contributary event	yes	2	14
	no	14	9
	missing	0	6
Provoking factors	rest	3	13
	action	4	1
	supine position	0	10
Distribution	face	6	2
	proximal	3	28
	distal	11	1
	proximal & distal	2	0
Distractibility during clinical examination	Yes/no	1/15	21/8

Electrophysiological characteristics			
Type of jerks	positive	12	29
	negative	0	0
	both	4	0
Burst duration (ms)	30–50	1	0
	50–100	14	0
	100–300	0	9
	>300	0	6
	variable	1	14
Distribution	focal	3	0
	multi focal	12	8
	segmental	1	1
	generalized	0	0
	variable	0	20
Back-averaging	number of jerks (median + IQR)	106 (36)	47 (323)
	CS present	7	0
	CS absent	9	0
	BP present	0	14
	BP absent	0	15

### 3.2 Bereitschaftspotential

**Fig. 1:**
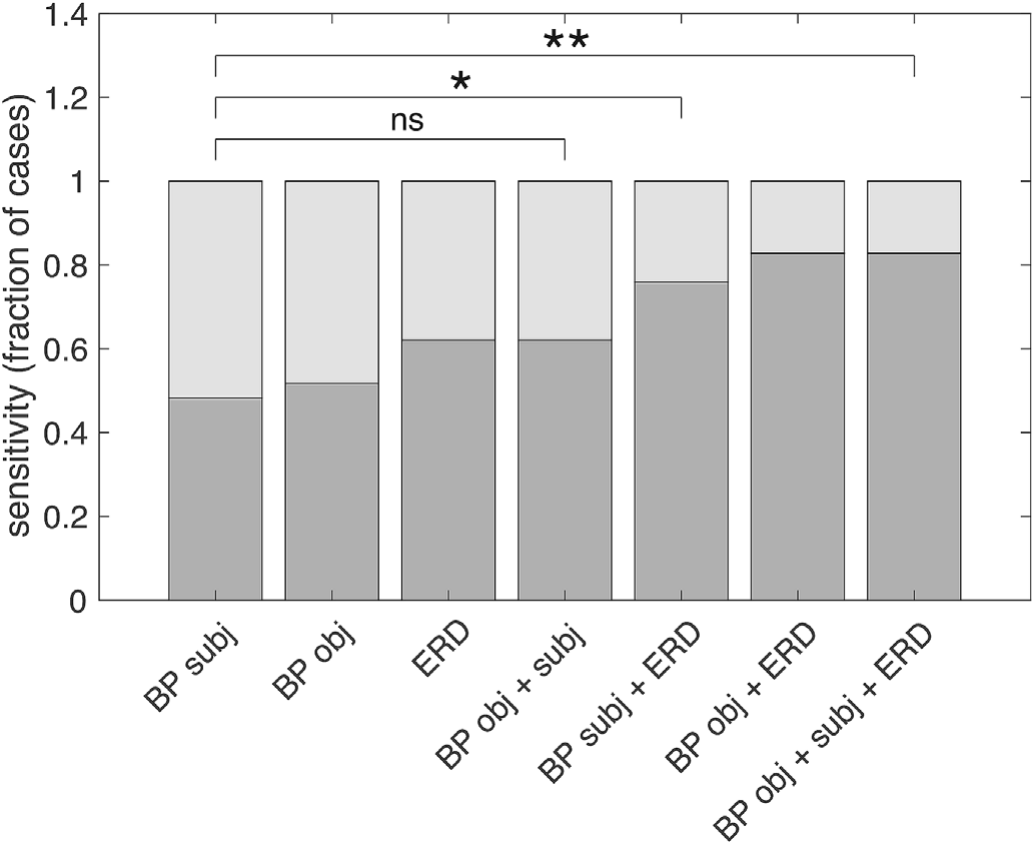
Comparison between the sensitivity of the visually determined BP (BP subj; subjective), the quantitatively determined BP (BP obj; objective) and event-related desynchronisation (ERD) and their combinations in ascending order. The sensitivity is depicted by the dark-grey bars which depict the fraction of patients in which neurophysiological evidence for a functional genesis of the myoclonic jerks is present, and vice versa. * = *p* < 0.05, ** = *p* < 0.01, ns = non-significant.

**Fig. 2:**
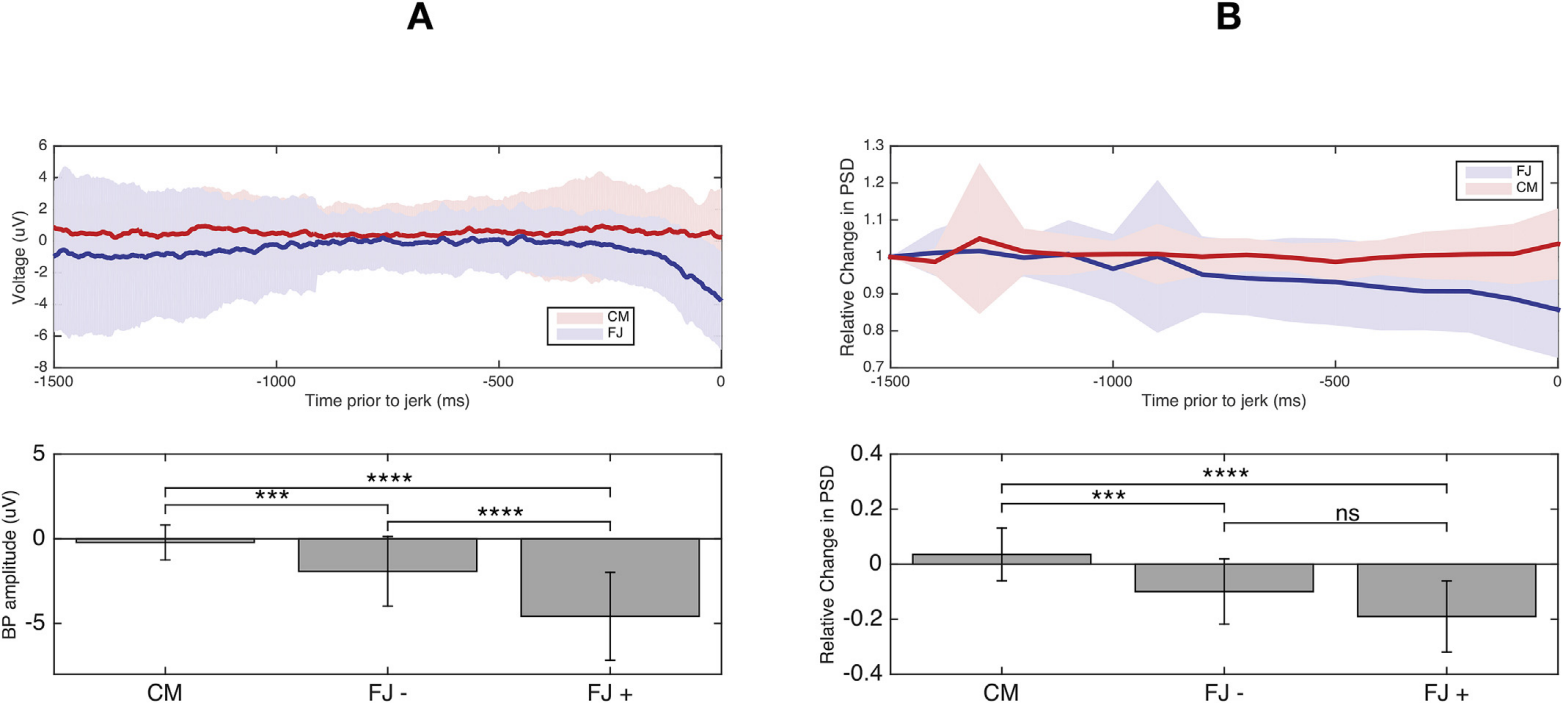
A: (*upper panel*) Time courses of central (Cz) EEG amplitude deflection prior to myoclonic jerks in the patient group with cortical myoclonus (CM) and functional myoclonic jerks (FJ) and their standard deviations ranging from −1500 ms prior to jerk to jerk onset. (*lower panel*) Average amplitude of central (Cz) EEG deflection prior to myoclonic jerks from −500 prior to jerk to jerk onset in cortical myoclonus (CM), functional jerks with absent (FJ -) or present (FJ +) visually rated bereitschaftspotential. B: (*upper panel*) Time courses of normalised central (Cz) EEG 13–45 Hz power spectral density (PSD) prior to myoclonus jerks (i.e. event related desynchronisation) in cortical myoclonus (CM) and functional jerks (FJ) and their standard deviations ranging from −1500ms prior to jerk to jerk onset. (*lower panel*) Average amplitude of normalised central (Cz) EEG 13–45 Hz power spectral density prior to myoclonic jerk from −1500 prior to jerk to jerk onset in cortical myoclonus (CM), functional jerks with absent (FJ -) or present (FJ +) visually rated bereitschaftspotential. μV = microvolt, ms = millisecond, *** = *p* < 0.005, **** = *p* < 0.001, ns = non-significant.

Using the subjective approach, a BP was present in 14/29 of the FJ patients and in none of the CM patients (sensitivity 47%; specificity 100%, Fig. 1). 15/29 (including 14 cases with a classical BP) had an objective BP that was lower than the lowest value of the CM group (i.e. - 2.18 V). This objective approach (‘BP obj’) had a sensitivity of 51% with a specificity of 100%. When comparing the average BP deflection of the subjective BP negative (n = 15, −1.91 ± 2.05 μV) and BP positive (n = 14, mean −4.75 ± 2.59 μV) FJ group with the CM group, differences in amplitude were statistically different (Average BP deflection (uV) respectively 6.2 (*p* < 0.001) and average BP deflection (uV) = 2.6 (*p* = 0.003), Fig. 2A.). Finally, when comparing the subjective BP negative with the BP positive group a significant difference was present within the FJ group as well (T = 5.1, *p* < 0.001).

### 3.3 Event related desynchronisation

FJ patients with or without a subjective BP both showed significantly more ERD in the broad beta band relative to CM (Fig. 2B, *p* = respectively < 0.001 and 0.001) and did not significantly differ from each other (*p* = 0.06). 18/29 FJ patients had an ERD that was lower than the lowest value of the CM group cut-off (i.e. 10% decrease in broad beta power). When using this 10% decrease as a differentiating criterion, a sensitivity of 62% was achieved with 100% specificity (Fig. 1). This did not significantly differ from using the objective BP approach (*p* = 0.62). No significant correlation was present between the number of jerks and ERD amplitude in either the CM or FJ group.

### 3.4 Relationship between ERD and objective BP

**Fig. 3:**
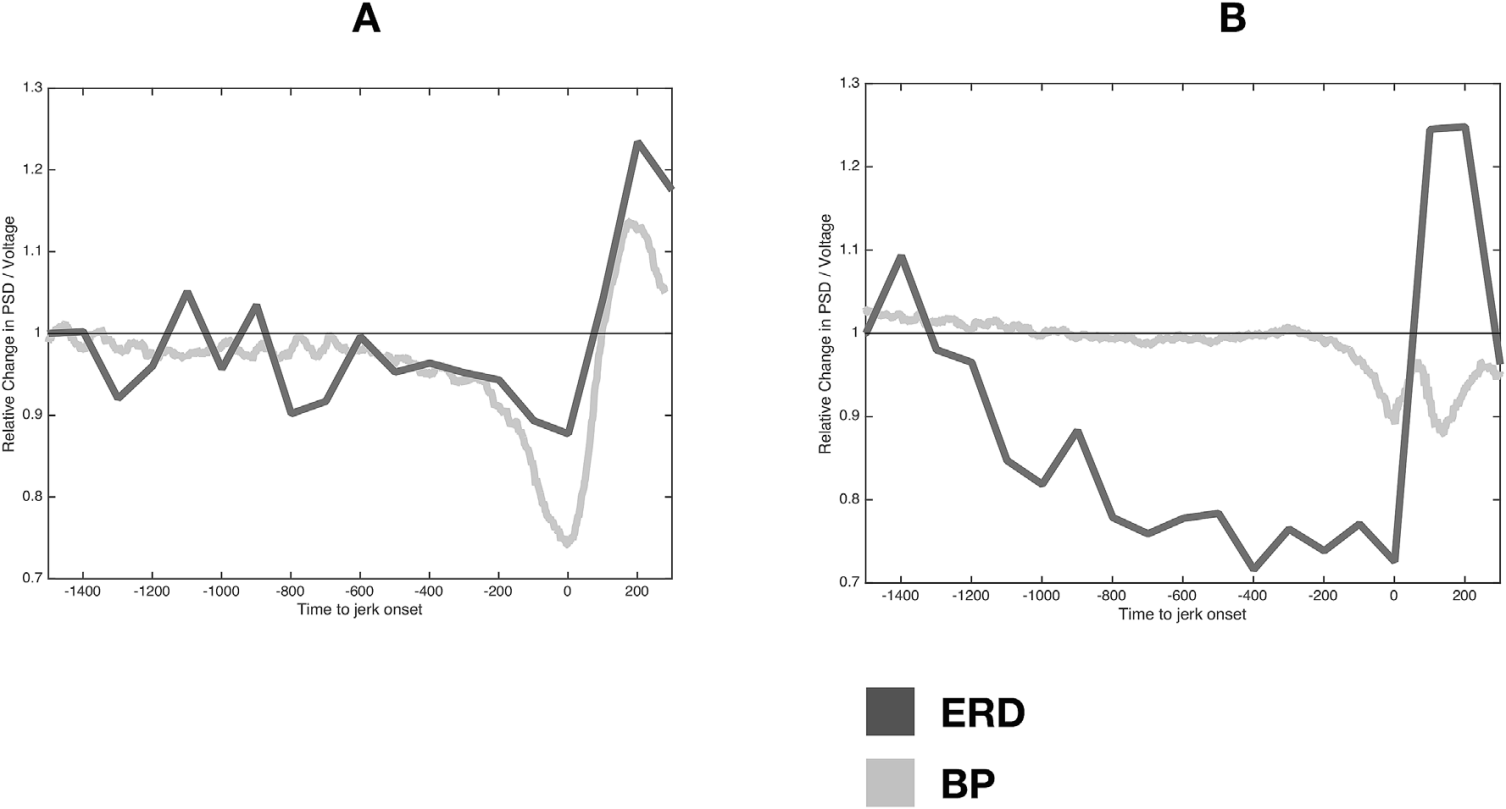
Examples of time courses of the Bereitschaftspotential (BP) and central (Cz) EEG 13–45 Hz Event Related Desynchronisation (ERD) prior to myoclonic jerks in two patients with functional jerks. A: simultaneous time-course of ERD and BP. B sequential time-course in which ERD starts earlier than BP that only consists of a late ‘negativity slope’ (see M & M).

**Suppl Fig. 1:**
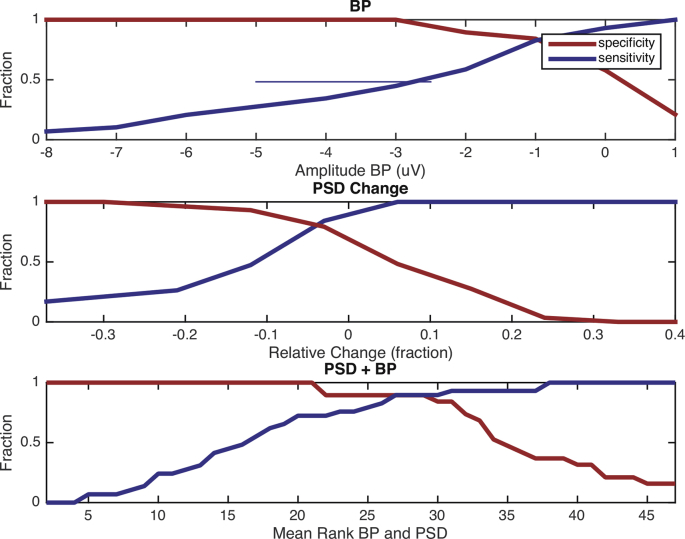
Correlation between the change in relative central (Cz) EEG 13–45 Hz power spectral density (13–45 Hz Change) prior to myoclonus jerks and the magnitude of the Bereitschaftspotential (BP) in cortical myoclonus (cortical, red dots) and functional jerks (functional, blue dots). The red line indicates the linear regression line for the cortical myoclonus group and the blue line the linear regression line for the functional myoclonus.

The amplitude of the objective BP and the ERD did not correlate significantly in the FJ (cc = 0.08, *p* = 0.67) or in the CM (cc 0.16, *p* = 0.53) group (Suppl Fig 1). An example of the temporal relation between the objective BP and ERD derived from two patients with FJ is provided in Fig. 3. In this figure it is visible that the BP and ERD can occur simultaneously (Fig. 3 A) or sequentially (Fig. 3 B).

### 3.5 Receiver operating characteristics

**Suppl Fig. 2:**
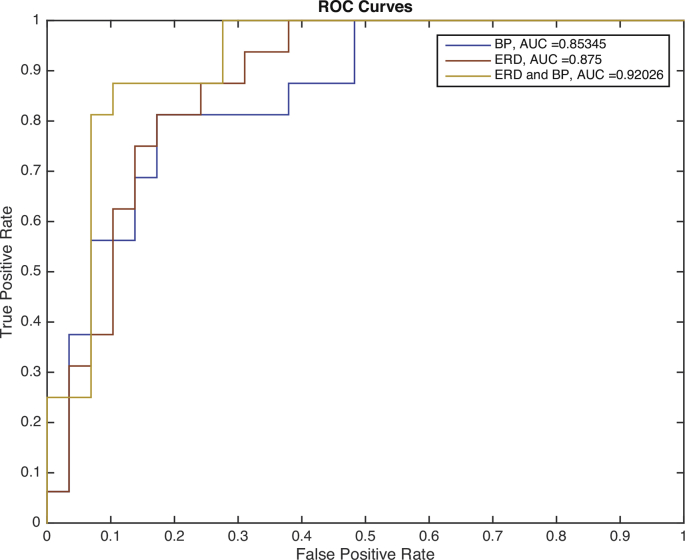
Receiver Operating Characteristics (ROC) for the use of the quantified Bereitschaftspotential (BP), Event Related Desynchronisation (ERD) and the combination of the two (ERD and BP) and their Areas Under the Curve (AUC, range 0–1). None of the AUC's differed significantly.

**Suppl Fig. 3:**
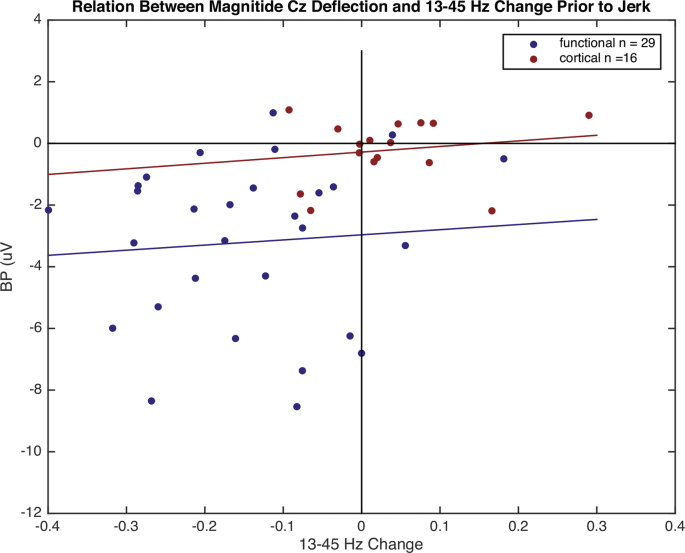
*Upper panel:* The relation between the amplitude of the quantified Bereitschaftspotential (BP) and its specificity (red) and sensitivity (blue, both in fractions) for discriminating functional jerks from cortical myoclonus. The horizontal bar indicate the values used in clinical practice for determining whether a BP is present which ranges from −5 μV to −2.5 μV. *Middle panel:* The relation between the change in relative central (Cz) EEG 13–45 Hz Power Spectral Density (PSD) prior to myoclonic jerks and its specificity and sensitivity for discriminating functional jerks from cortical myoclonus. *Lower panel:* The relation between the rank-based combination of BP and change in PSD and its specificity and sensitivity for discriminating functional jerks from cortical myoclonus. n.b. the rank is based on the average rank of BP and PSD in a cohort of 45 patients.

Both the objective BP and the ERD approach showed a ‘good’ (i.e. AUC between 0.8 and 0.9) ROC AUC (Suppl Fig 2). When combining the two methods, an ‘excellent’ (i.e. AUC between 0.9 and 1.0) ROC AUC was obtained. There was no statistically significant difference between objective BP and ERD analysis (*p* = 0.66). This was also the case when comparing the objective BP and ERD separately with their combination (i.e. obj BP + ERD, *p* respectively, 0.52 and 0.29). In Supplementary Fig. 3 the relations between sensitivity and specificity at different voltage/relative power changes are presented.

### 3.6 Diagnostic gain

When using the ERD prior to the myoclonic jerk, 8 of 15 with a FJ, that had a negative subjective BP, could be distinguished from CM without losing specificity (Fig. 1). This resulted in a diagnostic gain of 53% compared to subjective BP alone that had a sensitivity of 14/29 (47%). This difference was significant (*p* = 0.03), whereas when adding the objective BP to the subjective BP no significant increase in diagnostic gain was obtained (29%, *p* = 0.29). Finally, when both adding the objective BP and the ERD, the highest increase in diagnostic gain was obtained (71%, *p* < 0.01).

## 4 Discussion

In this study we were able to replicate the recent finding of the presence of event-related desynchronisation (ERD) in the broad (13–45 Hz) beta band preceding functional jerks (FJ) beyond the propriospinal myoclonus phenotype (e.g. focal, multi-focal and segmental FJ). In addition, we showed that its sensitivity for detecting a functional origin of myoclonus jerks is higher compared to the classical subjective BP. Furthermore, we showed that when the ERD method is added in BP negative patients a significant additional diagnostic gain of 53% is achieved. Finally, when adding a quantified, ‘objective’ BP analysis this gain increases to 71%. This meant that sensitivity (at 100% specificity) increased from 47% to 80%. All analyses were performed using a straightforward approach was in order to ease its applicability in clinical practice.

In the previous study on ERD in FJ, only patients with propriospinal FJ were included [18]. The current data show that beta ERD occurs in all kinds of FJ phenotypes (Table 1). In addition, beta ERD was recently reported to occur prior to psychogenic non-epileptic seizures [19]. This suggest that beta ERD might be a useful diagnostic marker for a wider range of paroxysmal functional neurological disorders.

At present the BP is often defined as a negative deflection prior to movement, exceeding 5 μV [26]. Our data suggest, however, that a less stringent definition of the BP (<−2.5 μV) is justified, as 100% specificity persists for distinguishing FJ from CM. In earlier reports, ‘borderline’ BP's with an amplitude of lower than −2.5 μV were interpreted blinded from the clinical case by experienced neurophysiologists [23]. Based on amplitude, shape, artifact and signal to noise ratio it was decided whether the BP was present or not in the study by van der Salm et al. In the study from van der Salm et al., as well as in our study, this resulted in an increase of the presence of BP's in FJ [23].

Interestingly, we found that the amplitude of ERD and BP were not correlated at the within subject level. Pathophysiologically, this might imply a different basis of these biomarkers. A previous study showed additional topographic segregation between BP and ERD, the latter being more widely distributed across temporal, parietal and higher-order motor area [22]. This is consistent with the idea that modulation of beta oscillations is related to attention [15]. Changes within attentional networks, reflected by ERD, are also predicted by the attention based model of functional neurological disorders [7]. The BP is mainly present in (pre)motor areas and might be a more direct reflection of the planned movement, although explanations are still speculative [1]. Both processes, i.e. altered attention and changes in planning of movement, are hypothesised to be disturbed in FJ.

### 4.1 Limitations

Our results might have been more pronounced in a selection of patients with identical jerks in the same body area [9]. However, the presence of ERD in our heterogeneous cohort demonstrates its potential applicability as a neurophysiological biomarker in a broader range of functional neurological disorders. Furthermore, the amount of patients with FJ and a positive BP is higher in earlier studies [23]. However, these studies had a prospective design and we cannot rule out that in our retrospective cohort neurophysiology was omitted in patients with sufficient clinical evidence for a functional origin of the jerks. The absence of a gold standard could have led to a misclassification of FJ and/or CM cases. However, in order to minimize this potential bias, we only included FJ and CM cases with both clinical *and* electrophysiological compatible findings [13,[25], [26], [27]]. Due to the demographic differences between FJ and CM, the median age differed between both groups (respectively mean age of 51 versus 28 years). However, we expect similar outcomes in younger FJ patients as previous studies reported no difference or a decrease of amplitude of the BP and a longer duration of ERD in elderly subject [6,21]. Furthermore, we only compared FJ with CM and not with other forms of organic myoclonus, e.g. subcortical myoclonus. For this reason we can't directly extrapolate our findings to all organic forms of myoclonus. The ‘excellent’ (AUC 0.9–10) ROC characteristics that were achieved by combining ERD and BP in a single cohort. We cannot prove with this study generalizability of our results, nevertheless this is the second cohort in which these ERD changes have been found [18].

In conclusion, ERD appears to be a promising neurophysiological biomarker alongside an accurate clinical examination, in the classification of FJ, especially in combination with objective BP. The reduction in beta oscillations prior to FJ found in our cohort strengthens the hypothesis of the role of changes within attentional networks in the pathogenesis of functional disorders. These findings stimulate further research regarding the applicability of ERD in clinical practice, pathophysiology of functional movement disorders, and exploration of therapeutic options influencing the beta power in FJ. Based on the current findings we propose adding ERD and objective BP analyses to the diagnostic algorithm for patients with a clinical suspicion of FJ with a negative subjective BP.

## Funding

S Little is supported by the Wellcome Trust (105804/Z/14/Z).
